# Fermented cotton stalks preserve colonic epithelial integrity in Hu sheep via the microbiota–metabolite–NF-κB/MLCK axis and mitigate the adverse effects of direct feeding

**DOI:** 10.3389/fnut.2026.1777023

**Published:** 2026-04-07

**Authors:** Peng Yang, Yaqi Meng, Yi Ma, Mengsi Xu, Xin Zhang

**Affiliations:** 1 Xinjiang Academy of Agricultural and Reclamation Sciences, Shihezi, Xinjiang, China; 2 School of Life Sciences, Jiangsu University, Zhenjiang, China

**Keywords:** butyrate, colonic barrier, fermented cotton stalk, Hu sheep, microbiota, NF-κB/MLCK

## Abstract

**Background:**

This study aimed to compare three cotton-stalk processing strategies—grinding (FS), steam explosion (PH), and microbial fermentation (FJ)—and to clarify whether fermented cotton stalks preserve colonic epithelial integrity through a microbiota–metabolite–NF‑κB/MLCK axis in Hu sheep.

**Methods:**

Fifteen clinically healthy Hu sheep (26.7 ± 1.76 kg body weight; 115 ± 4 days of age) were used after a 14‑day adaptation period and randomly assigned to one of three diets (*n* = 5 per treatment) containing 40% processed cotton stalks (FS, PH, or FJ) for 8 weeks.

**Results:**

PH and FJ increased final body weight compared with FS, and average daily gain increased progressively from FS to PH to FJ (206.07, 282.50, and 322.14 g/d, respectively; *p* < 0.05). Colonic fermentation profiles were markedly improved by FJ, evidenced by lower pH, ammonia nitrogen, free gossypol, and acetate (*p* < 0.05), alongside higher total VFAs with elevated propionate and butyrate (*p* < 0.05), whereas LPS was not different among treatments (*p* > 0.05). Histology and scanning electron microscopy indicated that FJ maintained intact crypt architecture and epithelial surface continuity, while FS exhibited epithelial detachment and surface erosion. Metagenomic analysis revealed distinct community structures among groups, with FJ showing higher richness and enrichment of taxa associated with carbohydrate utilization and butyrate‑producing guilds (e.g., *Lachnospiraceae*‑related genera such as *Anaerostipes*, *Blautia*, and *Coprococcus*). Consistently, FJ suppressed colonic mucosal inflammation, as reflected by reduced IL‑1β, IL‑6, IL‑8, and TNF-*α* at both mRNA and protein levels (*p* < 0.05). Mechanistically, FJ attenuated NF‑κB activation and downstream MLCK signaling, shown by decreased p‑p65/p65, p‑IκB/IκB, MLCK abundance, and p‑MLC/MLC ratio (*p* < 0.05), while upregulating tight‑junction proteins (ZO‑1, occludin, claudin‑1, and claudin‑4; (*p* < 0.05).

**Conclusion:**

Fermentation‑based processing of cotton stalks enhanced growth performance and promoted a favorable hindgut fermentation and microbial–metabolic milieu, thereby reinforcing colonic barrier integrity via inhibition of NF‑κB/MLCK‑associated inflammatory signaling, supporting fermented cotton stalk as a practical strategy to valorize cotton residues for ruminant feeding while mitigating gossypol‑related hindgut stress.

## 1 Introduction

Cotton stalks are the major by-products of cotton production, rich in lignocellulosic complexes and containing a measurable level of free gossypol (FG) (1). Their utilization as feed for ruminants could alleviate forage shortages, lower feeding costs, and enhance the value-added use of agricultural residues (2). However, the dense lignocellulosic structure and lignin encapsulation limit microbial accessibility and nutrient release (50), while FG exerts potential toxic effects that can induce mucosal stress and inflammatory responses, impairing gut health and animal performance (3, 4). Hence, developing appropriate processing technologies that simultaneously overcome structural barriers and mitigate FG toxicity is essential for the safe and efficient use of cotton stalks in ruminant diets. Common strategies include physical grinding (FS) to reduce particle size, steam-exploded cotton stalks (PH) to alter hemicellulose and starch structures, and microbial fermentation (FJ) using lactic acid bacteria and yeasts (5, 6), which can acidify substrates, generate enzyme systems, improve palatability, and partially decrease antinutritional factors.

In the ruminant large intestine, colonic fermentation end-products and nitrogenous metabolites jointly shape the local physicochemical milieu and provide energy to epithelial cells (7). Among volatile fatty acids (VFAs), butyrate plays a pivotal role in maintaining tight-junction integrity and promoting epithelial repair (8). The barrier function of the intestinal epithelium depends on the coordination of tight-junction and adherens-junction proteins, which are highly sensitive to inflammatory and cytoskeletal regulation (9). Activation of the NF-κB pathway elevates pro-inflammatory cytokines and weakens tight-junction integrity, while myosin light-chain kinase (MLCK) activation increases phosphorylation of myosin light chain (MLC), enhances actomyosin contraction, and enlarges intercellular spaces, resulting in increased permeability (10, 11). Growing evidence suggests that alterations in the intestinal microbiota and metabolite profiles can inhibit NF-κB activation, down-regulate the MLCK–p-MLC axis, and up-regulate tight-junction proteins, thereby reinforcing epithelial integrity (12). Nevertheless, systematic evidence linking “cotton-stalk processing mode-colonic microecology/metabolism - NF-κB/MLCK signaling - barrier phenotype” remains limited, particularly studies integrating fermentation products, FG residues, and multi-omics (metagenomic and metabolomic) analyses.

Although previous reports have demonstrated that extrusion and other physical pretreatments can improve lignocellulose accessibility (13), and that targeted microbial fermentation effectively reduces FG (48), comprehensive evaluations comparing the three major processing strategies—FS, PH, and FJ—within the same animal model are still lacking. This study aims to compare the effects of FS, PH, and FJ on the colonic microbiota–metabolite–NF-κB/MLCK–epithelial barrier axis in sheep, and to determine whether fermentation processing better maintains colonic epithelial integrity while mitigating the potential adverse effects of direct feeding.

## 2 Materials and methods

### 2.1 Animals and experimental procedures

The experimental design and animal management protocol were reviewed and approved by the Animal Care and Use Committee of the Xinjiang Academy of Agricultural and Reclamation Sciences (Xinjiang, China), and all procedures complied with its guidelines for the care and use of experimental animals.

A total of 15 clinically healthy Hu sheep with an initial body weight of 26.7 ± 1.76 kg and an average age of 115 ± 4 days were used in this study. All sheep were born to ewes of parity 1–2. Before the start of the trial, sheep were orally drenched with ivermectin (0.2 mg/kg BW) to eliminate endoparasites. Thereafter, the sheep were subjected to a 14-day adaptation period to the experimental housing and basal diets. At the end of the adaptation period, the sheep were randomly assigned, based on body weight, to one of three dietary treatments (*n* = 5 per treatment): (1) a diet containing chopped cotton stalks (FS); (2) a diet containing steam-exploded cotton stalks (PH); or (3) a diet containing fermented cotton stalks (FJ). The experimental feeding period lasted for 8 weeks.

Throughout the experiment, sheep were housed individually in pens (1.3 × 1.3 m) equipped with individual feed troughs and automatic drinkers, and had free access to fresh water. The ingredient and nutrient composition of the diets is shown in Table 1. Chinese wildrye (*Elymus dahuricus*) was used as the main forage, to which cotton stalks processed according to the respective treatment (FS, PH, or FJ) were added, together with a concentrate supplement formulated to meet or exceed the nutrient requirements of growing Hu sheep. Sheep were fed twice daily at 0800 and 1800 h. For each feeding, forage (Chinese wildrye plus the corresponding form of cotton stalk) was offered first, and concentrate was supplied after most of the forage had been consumed. The amount of feed offered was adjusted daily according to the previous day’s refusals, with the aim of maintaining roughage refusals at approximately 5–10% of the amount offered.

**Table 1:** Nutrient composition and ingredients of the experimental diets.

Item^1^	FS	PH	FJ
Corn	31.0	32.0	32.0
Cottonseed meal	17.0	15.5	15.0
Safflower seed meal	6.0	6.5	7.0
Corn stover	0	0	0
Ground cotton stalk	40.0	0	0
Steam-exploded cotton stalk	0	40.0	0
Fermented cotton stalk	0	0	40.0
Dicalcium phosphate	0.9	1.2	0.9
Limestone	1.4	1.1	1.4
Salt	0.7	0.7	0.7
2% premix^2^	3.0	3.0	3.0
Total	100	100	100
Nutrient levels			
Digestible energy (MJ/kg)	11.92	11.94	12.29
Crude protein (%)	14.15	14.13	14.18
NDF (%)	41.9	38.23	36.22
Ca (%)	0.93	0.89	0.95
P (%)	0.49	0.54	0.53

^1^FS, diet containing ground cotton stalks; PH, diet containing steam-exploded cotton stalks; FJ, diet containing fermented cotton stalks. ^2^The 2% premix supplied the following per kilogram of complete diet: 6.00 × 10^3^ IU vitamin A, 3.00 × 10^3^ IU vitamin D, 85.0 mg vitamin E, 6.30 mg Cu, 70.0 mg Fe, 65.0 mg Zn, 48.0 mg Mn, 0.130 mg I, 0.120 mg Co, and 0.120 mg Mo.

Cotton stalks used in the FS treatment were first air-dried under ambient conditions and manually cleaned to remove soil and other foreign materials. The cleaned stalks were then processed in two steps: they were initially cut into short pieces using a forage chopper, and subsequently ground in a hammer mill equipped with a 6–8 mm screen, resulting in stalk particles with a target length of approximately 6–8 mm. This material was then used as part of the roughage for the FS group.

For the PH treatment, cotton stalks were processed by steam explosion. Briefly, air-dried stalks were adjusted to a moisture content of approximately 35–40%, loaded into a batch steam-explosion reactor, and treated with saturated steam at about 130 °C (0.25–0.30 MPa) for 2–3 min. At the end of the residence time, the reactor pressure was released instantaneously to atmospheric pressure, causing rapid decompression and fiber expansion and partially disrupting the lignocellulosic structure. The exploded material was then spread in a thin layer, allowed to cool, air-dried to a moisture content close to the original level, and ground through a 6–8 mm screen to obtain a particle size comparable to that of the FS material before being offered to the PH group.

Cotton stalks used in the FJ treatment were first chopped to a particle length of approximately 6–8 mm as described for the FS group and then preserved by anaerobic fermentation. Freshly processed stalks were adjusted to a dry matter content of about 35–40% and uniformly sprayed with a mixed microbial inoculant containing *Lactiplantibacillus plantarum* ATCC 14917 (2.0 × 10^7^ cfu/kg fresh matter), *Saccharomyces cerevisiae* ATCC 18824 (2.5 × 10^7^ cfu/kg), *Enterococcus lactis* DSM 7134 (2.0 × 10^7^ cfu/kg), *Lactococcus lactis* ATCC 11007 (1.0 × 10^6^ cfu/kg), and *Candida utilis* ACCC 20060 (2.0 × 10^6^ cfu/kg). The treated material was then tightly packed into plastic-lined bales, compacted (by vacuum or manual pressing) to expel as much air as possible, and wrapped with several layers of stretch film to ensure anaerobic conditions. The bales were stored indoors at ambient temperature (approximately 15–25 °C) for at least 45 days before opening and feeding to the FJ group.

Feed offered and refusals were recorded daily for each sheep. On each sampling day, small subsamples (approximately 100 g) of the forage portion of the diet (Chinese wildrye plus the corresponding processed cotton stalk) and of the refusals were collected from each animal. Subsamples within each treatment were thoroughly mixed and immediately stored at −20 °C. At the end of the experiment, equal portions of the frozen subsamples were pooled by treatment to obtain composite, representative samples for subsequent chemical analyses. On the final day of the 8-week trial, the morning feeding at 0800 h was designated as the last meal for all sheep. Thereafter, all animals were transported to a local abattoir and slaughtered between 1200 and 1300 h (i.e., 4 ± 0.5 h after the last feeding) for collection of colonic tissues and digesta.

### 2.2 Sample collection

On the final day of the 8-week trial, sheep were slaughtered and samples from the gastrointestinal tract were collected within approximately 4 h after the last feeding. After opening the abdominal cavity along the midline, the middle segment of the colon (about 20–30 cm distal to the caecum) was rapidly located, ligated at both ends, and a 15–20 cm section was excised for subsequent sampling.

Colonic epithelial tissues were collected as follows. The colon segment was opened longitudinally and gently rinsed with ice-cold sterile physiological saline to remove luminal contents, and the surface was blotted dry with filter paper. Tissue blocks (approximately 0.5–1.0 cm^2^) for histomorphological examination were immediately fixed in 4% paraformaldehyde at 4 °C for at least 24 h, and later processed for paraffin embedding and hematoxylin–eosin (H&E) staining for light microscopy. Additional epithelial tissue pieces of similar size were placed in pre-cooled 2.5% glutaraldehyde at 4 °C for fixation and subsequent scanning electron microscopy. For molecular and inflammatory analyses, colonic mucosal epithelium was carefully sampled on an ice-cold plate, rapidly transferred into pre-chilled sterile tubes, snap-frozen in liquid nitrogen, and stored at −80 °C until determination of epithelial mRNA and protein expression and inflammatory cytokine levels.

For collection of colonic digesta, the ligated colon segment was cut open and one end was positioned over a sterile beaker while the contents were gently squeezed out from the other end. The digesta were then passed through four layers of sterile gauze, and the resulting filtrate was thoroughly mixed; its pH was measured immediately using a high-precision handheld pH meter (Sartorius, Goettingen, Germany), after which the filtrate was aliquoted according to the planned analyses. Aliquots of the filtrate intended for assessment of colonic fermentation characteristics and for measurement of LPS, free gossypol and related indices were dispensed into centrifuge tubes, pretreated as required for the respective assays, and immediately stored at −20 °C or −80 °C. For microbiota (metagenomic) and metabolomic analyses, approximately 2–5 g of fresh colonic digesta (unfiltered or only lightly filtered to remove coarse particles) were collected into sterile cryovials, snap-frozen in liquid nitrogen and stored at −80 °C until DNA extraction and metabolite profiling.

### 2.3 Histological observations

#### 2.3.1 H&E staining and light microscopy

Colonic tissue samples fixed in 4% paraformaldehyde were dehydrated in a graded ethanol series, cleared in xylene, and embedded in paraffin according to standard procedures (14). Paraffin blocks were sectioned at approximately 4–5 μm thickness using a rotary microtome and mounted on glass slides. After drying, sections were deparaffinized in xylene, rehydrated through graded ethanol to distilled water, and stained with hematoxylin followed by eosin. The stained sections were then dehydrated, cleared, and coverslipped with a neutral mounting medium. Colonic mucosal morphology, including epithelial continuity, crypt architecture, and inflammatory cell infiltration, was examined under a light microscope (IXplore IX85, Olympus Corporation, Japan) at appropriate magnifications, and representative images were captured with a digital imaging system.

#### 2.3.2 Scanning electron microscopy

Colonic epithelial specimens fixed in 2.5% glutaraldehyde were rinsed three times in 0.1 mol/L phosphate buffer (pH 7.4) and post-fixed in 1% osmium tetroxide for 1–2 h at 4 °C. Samples were then dehydrated through a graded ethanol series (30, 50, 70, 80, 90, 95, and 100%), transferred to isoamyl acetate, and dried using a critical-point dryer (15). The dried tissues were mounted on aluminum stubs, sputter-coated with a thin layer of gold, and observed with a scanning electron microscope (Apreo ChemiSEM, Thermo Fisher, USA) operated at an appropriate accelerating voltage. The surface morphology of the colonic epithelium, including microvilli integrity and intercellular junctions, was recorded by digital micrographs for subsequent qualitative evaluation.

### 2.4 Determination of LPS, free gossypol, ammonia nitrogen, and volatile fatty acids in colonic digesta

Colonic digesta samples collected as described above were thawed on ice and centrifuged at 10,000 × g for 10 min at 4 °C to obtain colonic fluid. The supernatant was harvested and used for the determination of fermentation parameters and LPS.

Individual and total volatile fatty acids (VFA) were determined by gas chromatography. Briefly, an aliquot of the colonic supernatant was mixed with metaphosphoric acid (25%, vol/vol), kept on ice for protein precipitation, and then centrifuged. The clarified supernatant was injected into a gas chromatograph (GC-14B, Shimadzu, Kyoto, Japan) equipped with a flame ionization detector, and individual VFAs were quantified according to the method of Lin et al. (16).

Ammonia nitrogen (NH₃–N) concentration in colonic fluid was measured colorimetrically using a commercial assay kit (Nanjing Jiancheng Bioengineering Institute, Nanjing, China) following the manufacturer’s instructions. Absorbance was read on a microplate reader (BioTek Instruments, Winooski, VT, USA) at the recommended wavelength, and NH₃–N concentrations were calculated from a standard curve prepared with ammonium chloride.

Free LPS in colonic fluid was quantified using a chromogenic end-point tachypleus amebocyte lysate assay (Chinese Horseshoe Crab Reagent Manufactory Co., Ltd., Xiamen, China). Briefly, supernatant samples were appropriately diluted with pyrogen-free water, mixed with the chromogenic reagent, and incubated according to the manufacturer’s protocol. The reaction was terminated at the specified time, and absorbance was measured with a microplate reader (BioTek Instruments, Winooski, VT, USA). All samples and standards were analyzed in duplicate, and LPS concentrations were calculated from the standard curve.

Free gossypol concentration in colonic fluid was determined using a colorimetric method after organic solvent extraction. In brief, an aliquot of the supernatant was mixed with an acetic acid–acetone extraction solution, and free gossypol in the extract was reacted with aniline to form a gossypol–aniline complex. After incubation in the dark for color development, absorbance was measured at the appropriate wavelength with a microplate reader (BioTek Instruments, Winooski, VT, USA), and free gossypol concentrations were calculated from a calibration curve prepared with gossypol standard solutions. All measurements were performed in duplicate.

### 2.5 Metagenomic sequencing

Colonic digesta samples used for metagenomic sequencing were obtained from the −80 °C stocks described above. For each sheep, approximately 2–5 g of frozen colonic digesta were thawed slowly on ice, thoroughly homogenized, and a portion was immediately taken for microbial genomic DNA extraction. Total microbial DNA was extracted using a commercial kit suitable for complex intestinal contents (QIAamp PowerFecal DNA Kit, QIAGEN, Hilden, Germany) according to the manufacturer’s instructions. Throughout the extraction procedure, repeated freeze–thaw cycles and prolonged exposure to room temperature were avoided to minimize DNA degradation and exogenous contamination.

The concentration and purity of the extracted DNA were first assessed using a spectrophotometer (NanoDrop 2000, Thermo Fisher Scientific, Waltham, MA, USA) by measuring the A_260_/A_280_ ratio, and DNA integrity and degradation were evaluated by 1% agarose gel electrophoresis. Only samples with an A₂₆₀/A₂₈₀ ratio between 1.8 and 2.0, a clear main band without obvious smearing, and a DNA concentration meeting the minimum requirement for library construction were retained. Qualified samples were further quantified using a fluorescence-based assay (Qubit 2.0 fluorometer, Thermo Fisher Scientific) to ensure accurate input for library preparation.

Metagenomic libraries were prepared following the standard paired-end library construction protocol for Illumina high-throughput sequencing platforms. Briefly, genomic DNA was randomly fragmented to an average size of approximately 350 bp using an ultrasonic shearing device. The fragmented DNA was then subjected to end repair, 3′-end A-tailing, and ligation of Illumina sequencing adapters. Adapter-ligated fragments were size-selected with magnetic beads to enrich DNA within the desired insert-size range, followed by a limited number of PCR amplification cycles to enrich fragments with properly ligated adapters. The amplified libraries were purified again using magnetic beads to remove residual primers, adapters, and short fragments. Library concentration and insert-size distribution were evaluated using a fluorometer and a fragment analyzer (Agilent 2,100 Bioanalyzer, Agilent Technologies, Santa Clara, CA, USA). Only libraries with appropriate concentration, a sharp insert-size peak, and no obvious primer dimers were used for sequencing.

Metagenomic sequencing was performed on an Illumina high-throughput platform (Illumina NovaSeq 6,000, Illumina, San Diego, CA, USA) using a paired-end mode with a read length of 150 bp (PE150). Raw reads generated from sequencing were subjected to quality control using dedicated software to remove reads containing adapter contamination, reads with a high proportion of ambiguous bases (N), and low-quality reads with an average quality score below a predefined threshold. High-quality clean reads were obtained after filtering. To remove host-derived sequences, clean reads were further mapped against the sheep (*Ovis aries*) reference genome, and reads aligning to the host genome were discarded. The remaining unmapped reads were retained as putative microbial reads for downstream analyses.

For assembly and gene prediction, the host-filtered clean reads were *de novo* assembled using a metagenome-specific assembler to generate contigs. Contigs meeting predefined length and coverage criteria were retained for subsequent gene prediction. Open reading frames (ORFs) were predicted on these contigs using a gene prediction tool (Prodigal), yielding a set of genes for each sample. ORFs from all samples were then clustered at a given sequence identity (95%) and coverage threshold to construct a non-redundant gene catalog (gene set), thereby reducing redundancy and facilitating cross-sample comparisons. Finally, clean reads from each sample were mapped back to the non-redundant gene catalog, and gene abundance in each sample was calculated based on the number of mapped reads, gene length, and sequencing depth. Abundance values were normalized to generate a comparable gene abundance matrix across samples. This gene catalog and its corresponding abundance profiles were subsequently used for taxonomic and functional analyses.

### 2.6 Metabolomic analysis of colonic digesta

Untargeted metabolomic analysis of colonic digesta was outsourced to a commercial service provider (Wuhan Maiwei Metabolomics Biotechnology Co., Ltd., Wuhan, China). Colonic digesta samples that had been stored at −80 °C were transported to the company on dry ice and kept frozen throughout shipment.

For sample preparation, frozen colonic digesta were thawed slowly on ice and thoroughly homogenized. Approximately 100 mg of colonic digesta from each animal were weighed into pre-cooled centrifuge tubes, and a defined volume of ice-cold methanol–water mixture (70:30, v/v) containing internal standards was added. The mixture was vortexed vigorously and subjected to ultrasonic extraction in an ice–water bath to enhance the release of small-molecule metabolites, followed by incubation at −20 °C to precipitate proteins. The samples were then centrifuged at high speed (14,000 × *g*, 10–15 min, 4 °C), and the clear supernatants were carefully transferred to new tubes. To monitor instrument stability and analytical reproducibility, quality control (QC) samples were prepared by pooling equal aliquots from all individual supernatants and analyzed together with the experimental samples.

Prior to instrumental analysis, supernatants were either injected directly or evaporated to near dryness under a gentle stream of nitrogen gas and reconstituted in an appropriate solvent (50% methanol in water). After vortex mixing and brief centrifugation to remove residual particulate matter, the final extracts were transferred into autosampler vials for liquid chromatography–mass spectrometry (LC–MS) analysis. Metabolomic profiling was carried out on an ultrahigh-performance liquid chromatography system coupled to a high-resolution tandem mass spectrometer (UHPLC–MS/MS; Thermo Fisher Scientific), operated in both positive and negative electrospray ionization modes. Samples were separated on a reversed-phase analytical column using a binary gradient elution program consisting of an aqueous phase and an organic phase (acetonitrile or methanol), both typically containing 0.1% formic acid or another suitable modifier. Column temperature, flow rate, injection volume and gradient conditions were set according to the standard operating procedures of the service provider to ensure robust separation of small-molecule metabolites. Mass spectrometric data were acquired in full-scan mode combined with data-dependent MS/MS, with appropriate settings for scan range, spray voltage, capillary temperature and gas flows. QC samples were injected at regular intervals throughout the analytical sequence to evaluate retention time stability and signal drift.

Raw LC–MS data were processed by the service provider using dedicated software for peak detection, extraction, alignment, deconvolution and signal normalization. Metabolic features (defined by retention time and mass-to-charge ratio) with poor reproducibility or a high proportion of missing values were removed according to predefined criteria. Putative metabolite identification was performed by matching accurate masses, MS/MS fragmentation patterns and retention times against in-house and public metabolite databases. The resulting data matrix, consisting of annotated metabolites and their normalized signal intensities across all samples, was used for subsequent multivariate and univariate statistical analyses.

### 2.7 Determination of inflammatory cytokines in colonic mucosa

Inflammatory cytokines in colonic epithelial mucosa were quantified by enzyme-linked immunosorbent assay (ELISA). Frozen colonic mucosal samples (collected as described above) were thawed on ice, and approximately 80–100 mg of epithelial mucosa from each sheep were carefully scraped, weighed, and transferred into pre-cooled tubes. An appropriate volume of ice-cold phosphate-buffered saline (PBS, pH 7.4) containing a protease inhibitor cocktail was added, and the tissue was homogenized on ice using a mechanical homogenizer until a uniform suspension was obtained. The homogenates were centrifuged at 10,000–12,000 × *g* for 10–15 min at 4 °C, and the supernatants were collected for cytokine and protein determination. Total protein concentration in each supernatant was measured using a bicinchoninic acid (BCA) protein assay kit (Thermo Fisher Scientific, Waltham, MA, USA) according to the manufacturer’s instructions. Cytokine concentrations were subsequently normalized to total protein content.

The concentrations of interleukin-1β (IL-1β), interleukin-6 (IL-6), interleukin-8 (IL-8), and tumor necrosis factor-*α* (TNF-α) in colonic mucosal supernatants were determined using commercial ELISA kits specific for ovine cytokines (Nanjing Jiancheng Bioengineering Institute, Nanjing, China), strictly following the manufacturers’ protocols. Briefly, standards and appropriately diluted samples were added to the antibody-coated wells and incubated for the prescribed time. After washing, detection antibodies and enzyme-conjugated reagents were added sequentially, followed by incubation with chromogenic substrate solution. The color reaction was terminated by the addition of stop solution, and the optical density was read at 450 nm using a microplate reader (BioTek Instruments, Winooski, VT, USA). Standard curves were generated from serial dilutions of the supplied standards, and the concentrations of IL-1β, IL-6, IL-8, and TNF-*α* in the samples were calculated from the corresponding standard curves. All samples were analyzed in duplicate, and the intra- and inter-assay coefficients of variation were kept below 10% for all cytokines.

### 2.8 Real-time PCR analysis of gene expression

Total RNA was extracted from colonic epithelial mucosa using a Tissue Total RNA Isolation Kit V2 (Vazyme Biotech, Nanjing, China) according to the manufacturer’s instructions. Briefly, frozen mucosal scrapings were homogenized in lysis buffer on ice, and the lysates were cleared by centrifugation before loading onto the spin columns. An on-column DNase I digestion step was included to remove residual genomic DNA. The concentration and purity of RNA were assessed using a NanoDrop spectrophotometer (NC2000, Thermo Fisher Scientific, Waltham, MA, USA), and only samples with an A₂₆₀/A₂₈₀ ratio between 1.8 and 2.1 and an A₂₆₀/A₂₃₀ ratio ≥ 1.8 were used for downstream analyses. RNA integrity was evaluated by denaturing 1.2% (wt/vol) agarose gel electrophoresis and by determining the RNA integrity number (RIN) with a Bioanalyzer 2,100 system (Agilent Technologies, Santa Clara, CA, USA); only RNA samples with a RIN ≥ 7.0 were reverse-transcribed into cDNA. First-strand cDNA was synthesized from 1.0 μg of total RNA using a commercial reverse transcription kit (HiScript II 1st Strand cDNA Synthesis Kit, Vazyme Biotech) following the manufacturer’s protocol, with a combination of oligo(dT) and random primers to ensure efficient coverage of mRNA species. The resulting cDNA was diluted appropriately with nuclease-free water and stored at −20 °C until quantitative PCR (qPCR) analysis.

Primers for target genes related to epithelial barrier function and inflammation (IL-1β, IL-6, IL-8, and TNF-*α*) were designed using Primer-BLAST to generate amplicons of 80–200 bp, and, where possible, to span exon–exon junctions to minimize amplification of genomic DNA. All primers were synthesized by Sangon Biotech (Shanghai, China), and their sequences are listed in Table 2. Primer specificity was verified by agarose gel electrophoresis of PCR products and by melt-curve analysis showing a single, sharp peak for each primer pair. To select a suitable internal control, three candidate reference genes (ACTB, GAPDH, and HPRT) were evaluated using RefFinder, which integrates NormFinder, geNorm, and the comparative ΔCt method. This analysis indicated that ACTB and GAPDH exhibited the highest and most comparable expression stability across all samples. Based on these results and for consistency with previous studies, ACTB was selected as the reference (housekeeping) gene for normalization in the present study.

**Table 2:** Primers for quantitative real-time PCR.

Gene name^1^	Sequences (5′ → 3′)^2^	Product size (bp)	Gene ID
IL-1β	**F:** CCTGGAAGCCATTGCCAATG	129	NM_001009808.1
	**R:** GCGTCGTTCAGGATGCATTC		
IL-6	**F:** GCTGCTCCTGGTGATGACTT	80	NM_001009392.1
	**R:** TGCTTGGGGTGGTGTCATTT		
IL-8	**F:** AAGCTGGCTGTTGCTCTCTT	129	NM_001009401.2
	**R:** GGGGTGGAAAGGTGTGGAAT		
TNF-α	**F:** AGAAGGGAGATCGCCTCAGT	80	NM_001024860.1
	**R:** CCCAAAGTAGACCTGCCCAG		
ACTB	**F:** CTTCCAGCCTTCCTTCCTGG	180	NM_001009784.3
	**R:** GCCAGGGCAGTGATCTCTTT		
GAPDH	**F:** ACAGTCAAGGCAGAGAACGG	98	XM_060411593.1
	**R:** CCAGCATCACCCCACTTGAT		
HPRT	**F:** AACGACTGGCTCGAGATGTG	100	XM_015105023.3
	**R:** TCCAACAGGTCGGCAAAGAA		

^1^TNF-α = Tumor Necrosis Factor α; IL-1β = Interleukin-1beta; GAPDH = glyceraldehyde-3-phosphate dehydrogenase; HPRT1 = hypoxanthine phosphoribosyltransferase 1; ^2^F = forward; R = reverse. Bold letters in the table indicate: F = forward; R = reverse.

Quantitative real-time PCR was performed on a CFX96 real-time PCR detection system (Bio-Rad Laboratories, Hercules, CA, USA) using a SYBR Green qPCR Master Mix (Vazyme Biotech) in a final volume of 10–20 μL per reaction. A typical amplification program consisted of an initial denaturation at 95 °C for 30 s, followed by 40 cycles of 95 °C for 10 s and 60 °C for 30 s. At the end of each run, a melt-curve analysis from 65 °C to 95 °C was performed to confirm amplification specificity. A no-template control (NTC) was included for each gene in every run to monitor potential contamination or primer–dimer formation. For each animal and each gene, qPCR reactions were performed in technical triplicate, and the mean cycle threshold (Ct) value was used for subsequent calculations. Relative mRNA expression levels of target genes were calculated using the 2^-ΔΔCt^ method (51), with ACTB serving as the reference (housekeeping) gene and one experimental group designated as the calibrator.

### 2.9 Protein extraction and Western blot analysis

Total protein was extracted from colonic epithelial mucosa. Briefly, approximately 80–100 mg of frozen colonic mucosal scrapings from each sheep were thawed on ice and transferred into pre-cooled tubes containing ice-cold RIPA lysis buffer (Solarbio Biotech Co., Ltd., Beijing, China) supplemented with a protease and phosphatase inhibitor cocktail. Tissues were homogenized on ice using a mechanical homogenizer until complete lysis was achieved, and the lysates were incubated on ice for an additional 30 min with intermittent vortexing. The homogenates were then clarified by centrifugation at 12,000 × *g* for 20–30 min at 4 °C, and the supernatants containing total protein were carefully collected. Protein concentration in each sample was determined in triplicate using an Enhanced BCA Protein Assay Kit (Vazyme Biotech Co., Ltd., Nanjing, China) according to the manufacturer’s instructions. Aliquots of the protein extracts were stored at −80 °C until further analysis. For Western blotting, protein samples were mixed with 5 × loading buffer, denatured by heating at 95–100 °C for 5 min, and then cooled on ice. Equal amounts of protein (typically 40–60 μg per lane) were separated by sodium dodecyl sulfate–polyacrylamide gel electrophoresis (SDS–PAGE) on 10–12% resolving gels. Electrophoresis was carried out at a constant voltage (e.g., 60 V for stacking and then 100 V for resolving) until the dye front reached the bottom of the gel. Separated proteins were electrotransferred onto nitrocellulose membranes (Millipore, Billerica, MA, USA) using a wet-transfer system. After transfer, membranes were briefly rinsed in Tris-buffered saline with 0.1% Tween-20 (TBST) and blocked with 5% bovine serum albumin (BSA; Beyotime Biotechnology, Shanghai, China) in TBST for 1 h at room temperature (approximately 25 °C) to reduce nonspecific binding. Following blocking, membranes were incubated overnight at 4 °C with the appropriate primary antibodies diluted in 5% BSA/TBST. The primary antibodies used in this study included: NF-κB p65, phospho-NF-κB p65 (p-p65), IκBα, phospho-IκBα (p-IκBα), myosin light chain (MLC), phospho-MLC (MLC Ser19), myosin light chain kinase (MLCK/MYLK), zonula occludens-1 (ZO-1), claudin-1, claudin-4, occludin, and ACTB (loading control). All primary antibodies were used at a working dilution of 1:1,000, and were purchased from commercial suppliers (Cell Signaling Technology, Danvers, MA, USA).

After incubation with primary antibodies, membranes were washed three times (5–10 min each) with TBST and then incubated for 45–60 min at room temperature with a horseradish peroxidase (HRP)-conjugated secondary antibody (goat anti-rabbit IgG or goat anti-mouse IgG, as appropriate; Beyotime Biotechnology) diluted 1:1,000–1:5,000 in 5% BSA/TBST. Following secondary antibody incubation, membranes were washed again in TBST and the immunoreactive bands were visualized using an enhanced chemiluminescence (ECL) detection kit (Vazyme Biotech Co., Ltd.) according to the manufacturer’s protocol. Chemiluminescent signals were captured using a digital imaging system (LAS-4000, GE Healthcare Bio-Sciences AB, Uppsala, Sweden). The band intensity of each protein was quantified using Image-Pro Plus 6.0 (Media Cybernetics Inc., Rockville, MD, USA) or equivalent image analysis software. The expression levels of target proteins (p65, p-p65, IκBα, p-IκBα, MLC, p-MLC, MLCK, ZO-1, claudin-1, claudin-4, and occludin) were normalized to ACTB, which served as the internal loading control. For phosphorylated proteins, the ratio of phosphorylated to total protein (e.g., p-p65/p65, p-IκBα/IκBα, p-MLC/MLC) was also calculated. Final data were expressed as fold changes relative to the mean value of the control group.

### 2.10 Statistical analysis

All data are presented as means ± pooled standard error of the mean (SEM). Statistical analyses were performed using SPSS software (SPSS Inc., Chicago, IL, USA). For growth performance, colonic fermentation parameters, inflammatory cytokine concentrations, as well as gene and protein expression levels, differences among dietary treatments (FS, PH and FJ) were evaluated using one-way ANOVA with diet as the fixed effect. When a significant overall effect was detected, Tukey’s *post hoc* test was used for pairwise comparisons between groups. Prior to ANOVA, data were checked for normality and homogeneity of variances; variables that did not meet these assumptions were log-transformed or rank-transformed as appropriate before re-analysis. Statistical significance is indicated by asterisks as follows: *p < 0.05, **p < 0.01 and ***p < 0.001. A *p*-value < 0.05 was considered statistically significant.

For metagenomic data, downstream bioinformatic and statistical analyses were based on the non-redundant gene abundance matrix and the corresponding taxonomic and functional annotations. Alpha (*α*) diversity indices were calculated from gene- or species-level abundance tables, and rarefaction curves were generated to assess whether sequencing depth and coverage were sufficient. Beta (*β*) diversity was calculated using Bray–Curtis dissimilarity based on relative abundance data and visualized by principal component analysis (PCA) in R (R Foundation for Statistical Computing, Vienna, Austria) using the “vegan” package, in order to compare microbial community structures among dietary groups. Differences in overall community composition among treatments were further tested by permutational multivariate analysis of variance (PERMANOVA; “adonis” function in vegan) with 999 permutations.

For functional metagenomic analysis, predicted genes were aligned against public databases to obtain Gene Ontology (GO) terms and KEGG orthology (KO) annotations. Based on normalized gene abundance tables, differentially abundant genes between groups were identified using appropriate statistical approaches (DESeq2 or non-parametric tests combined with Benjamini–Hochberg multiple testing correction). GO term and KEGG pathway enrichment analyses were then performed on these sets of differentially abundant genes using a hypergeometric test. GO terms or KEGG pathways with a false discovery rate (FDR)–adjusted *p* < 0.05 were considered significantly enriched. To identify microbial taxa that were differentially enriched among dietary treatments, linear discriminant analysis effect size (LEfSe) was applied to the relative abundance data at different taxonomic levels. LEfSe combines the Kruskal–Wallis test, pairwise Wilcoxon rank-sum tests and linear discriminant analysis (LDA); taxa with *p* < 0.05 and LDA scores (log₁₀) ≥ 2.0 were regarded as discriminative biomarkers associated with specific diets.

For metabolomic data, peak intensities obtained from LC–MS analysis were first log-transformed and then Pareto-scaled for normalization. Unsupervised PCA was performed to evaluate clustering and separation among dietary groups, as well as within-group reproducibility and potential outliers. Differential metabolites between groups were identified based on a combination of fold change and univariate statistics: metabolites with an absolute log₂(fold change) ≥ 1 and an FDR-adjusted *p* < 0.05 (ANOVA) were defined as significantly different. Volcano plots were generated to visualize the distribution of differential and non-differential metabolites. Differential metabolites were further mapped to KEGG pathways, and KEGG pathway enrichment analysis was conducted using a hypergeometric test; pathways with FDR-adjusted *p* < 0.05 were considered significantly enriched.

## 3 Results

### 3.1 Effects of processed cotton stalk diets on growth performance of Hu sheep

As shown in Table 3, initial body weight did not differ among the three groups at the start of the experiment (*p* = 0.133), indicating a successful random allocation of sheep.

**Table 3:** Growth performance of Hu sheep fed diets containing differently processed cotton stalks.

^1^Item	FS	PH	FJ	SEM	*p*-value
Initial weight (kg)^2^	26.62^a^	27.06^a^	26.30^a^	0.61	0.133
Final weight (kg)	38.16^b^	42.88^a^	44.34^a^	0.99	0.046
Net weight gain (kg)	11.54^c^	15.82^b^	18.04^a^	0.81	0.013
ADG (g/d)	206.07^c^	282.50^b^	322.14^a^	16.38	0.006

^1^FS, diet containing ground cotton stalks; PH, diet containing steam-exploded cotton stalks; FJ, diet containing fermented cotton stalks. ADG, average daily gain. ^2^Values within a row with different superscript letters differ significantly (*p* < 0.05). Values are expressed as means (*n* = 5 per group); pooled SEM and overall diet effect *p*-values from one-way ANOVA are shown.

In contrast, final body weight was affected by dietary treatment (*p* = 0.046). Sheep fed the PH and FJ diets had greater final weights than those fed the FS diet (*p* < 0.05), whereas the difference between PH and FJ was not statistically significant (*p* > 0.05). Net weight gain and average daily gain (ADG) increased progressively from FS to PH to FJ (*p* = 0.013 and 0.006, respectively).

### 3.2 Fermented cotton stalks maintained the integrity of the colonic epithelium in sheep

**Figure 1:**
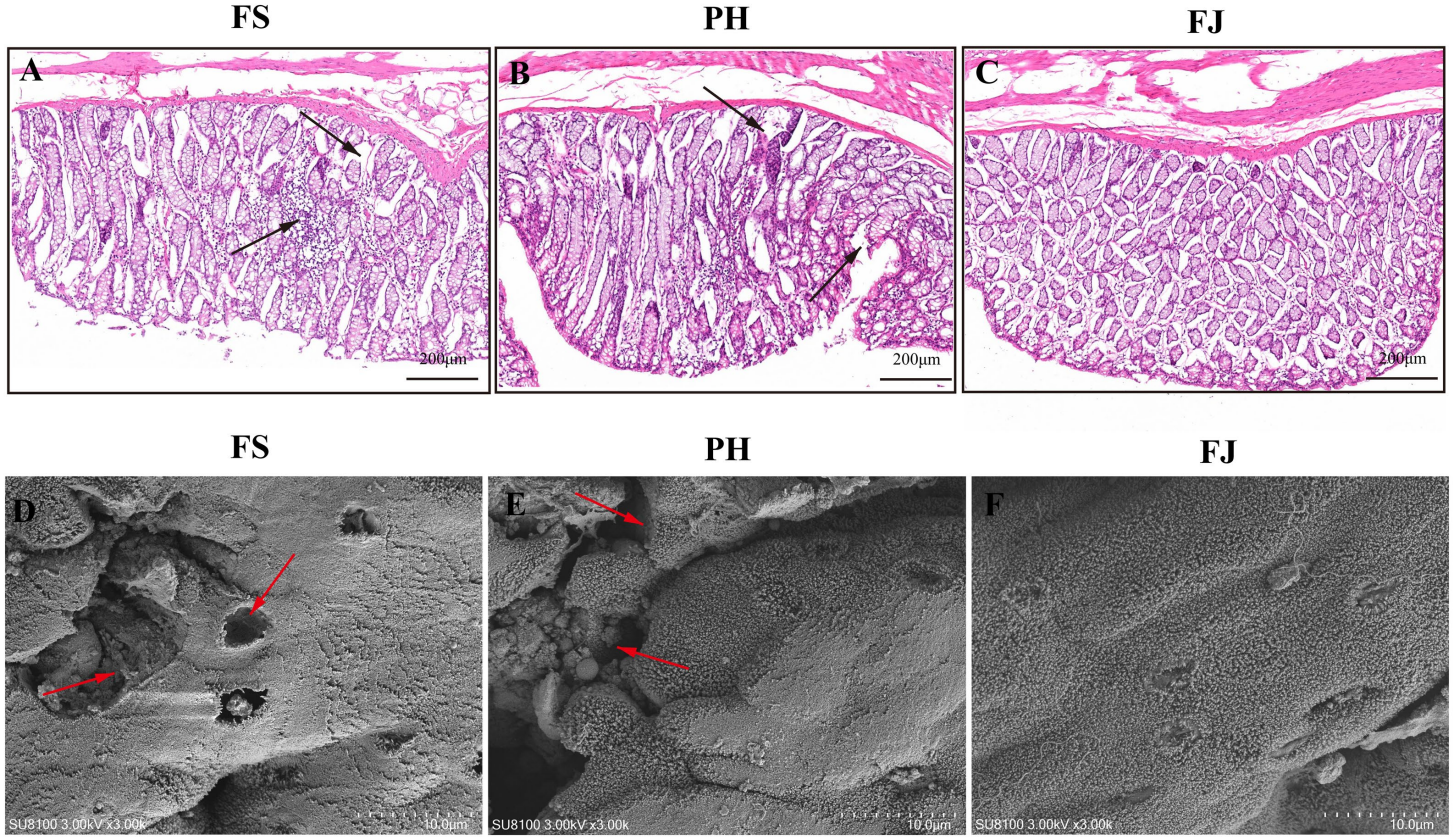
Fermented cotton stalks preserve colonic epithelial morphology and ultrastructure in Hu sheep. **(A–C)** Representative hematoxylin and eosin (H&E)–stained sections of the colonic mucosa from sheep fed diets containing FS, PH, or FJ diets. **(D–F)** Representative scanning electron microscopy (SEM) images showing the colonic epithelial surface microstructure in the FS, PH, and FJ groups. FS = diet containing ground cotton stalks; PH = diet containing steam-exploded cotton stalks; FJ = diet containing fermented cotton stalks; *n* = 5 sheep/treatment. Scale bars = 200 m **(A–C)** and 10 m **(D–F)**.

H&E observations revealed distinct differences in colonic morphology among the three dietary groups (Figure 1). In the FS group (Figure 1A), the colonic mucosa displayed irregular and loosely arranged crypts, with partial epithelial detachment and mild inflammatory cell infiltration in the lamina propria, indicating local barrier impairment. The PH group (Figure 1B) showed a partially improved epithelial arrangement compared with FS, yet crypt distortion and epithelial surface discontinuity were still evident. In contrast, the FJ group (Figure 1C) exhibited well-organized and intact crypt structures, with dense epithelial alignment and clear mucosal boundaries. The lamina propria was compact with minimal inflammatory infiltration, suggesting a more stable epithelial morphology.

Scanning electron microscopy (SEM) further confirmed these findings: the epithelial surface of the FS group appeared rough and uneven, showing multiple erosive pits and disrupted microstructures (Figure 1D). The PH group showed partial surface restoration, but still displayed shallow depressions and loosened cellular junctions (Figure 1E). Conversely, the FJ group retained a smooth, continuous epithelial surface with densely distributed microvilli and no visible erosions, indicating enhanced epithelial integrity and reduced structural damage (Figure 1F).

### 3.3 Feeding fermented cotton stalks increased the fermentative efficiency of colonic contents in sheep

**Figure 2:**
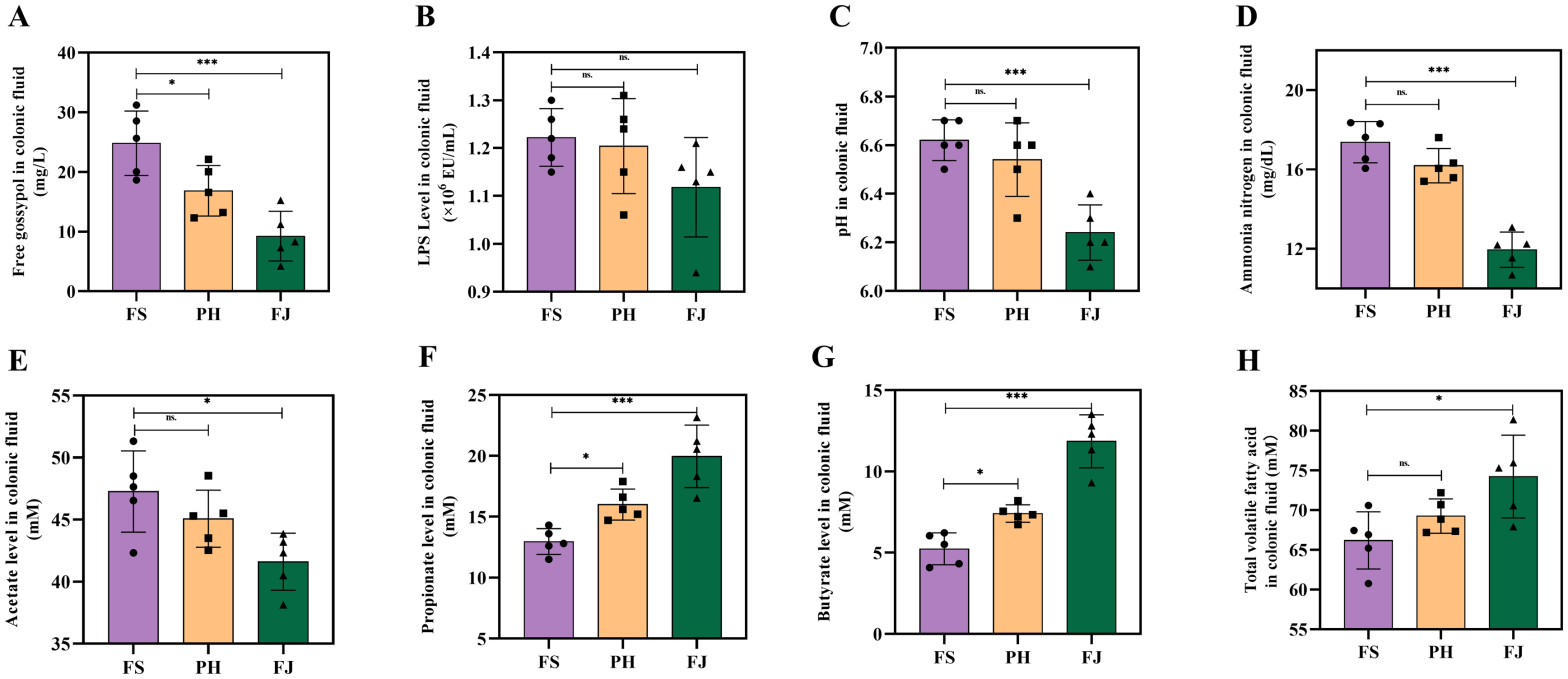
Effects of differently processed cotton stalk diets on colonic fermentation characteristics and luminal indices in Hu sheep. **(A)** Free gossypol concentration, **(B)** LPS level, **(C)** pH, **(D)** NH_3_–N, **(E)** acetate, **(F)** propionate, **(G)** butyrate, and **(H)** total volatile fatty acids (TVFA) in colonic fluid of sheep fed diets containing FS, PH, or FJ diets. FS = diet containing ground cotton stalks; PH = diet containing steam-exploded cotton stalks; FJ = diet containing fermented cotton stalks; *n* = 5 sheep/treatment. Data are presented as means ± SEM. Statistical differences among treatments are indicated by *p < 0.05, **p < 0.01 and ***p < 0.001; ns, not significant.

As shown in Figure 2, compared with the FS group, the PH group significantly reduced the concentration of free gossypol in colonic fluid (*p* < 0.05) and increased the concentrations of propionate and butyrate (*p* < 0.05). In addition, compared with the FS group, the FJ group markedly decreased the levels of free gossypol, pH, ammonia nitrogen, and acetate in colonic fluid (*p* < 0.001), while significantly elevating the concentrations of propionate, butyrate, and total VFAs (*p* < 0.05). No significant difference in LPS concentration was observed among the groups (*p* > 0.05).

### 3.4 Feeding fermented cotton stalks supported microbial homeostasis in the sheep colon

**Figure 3:**
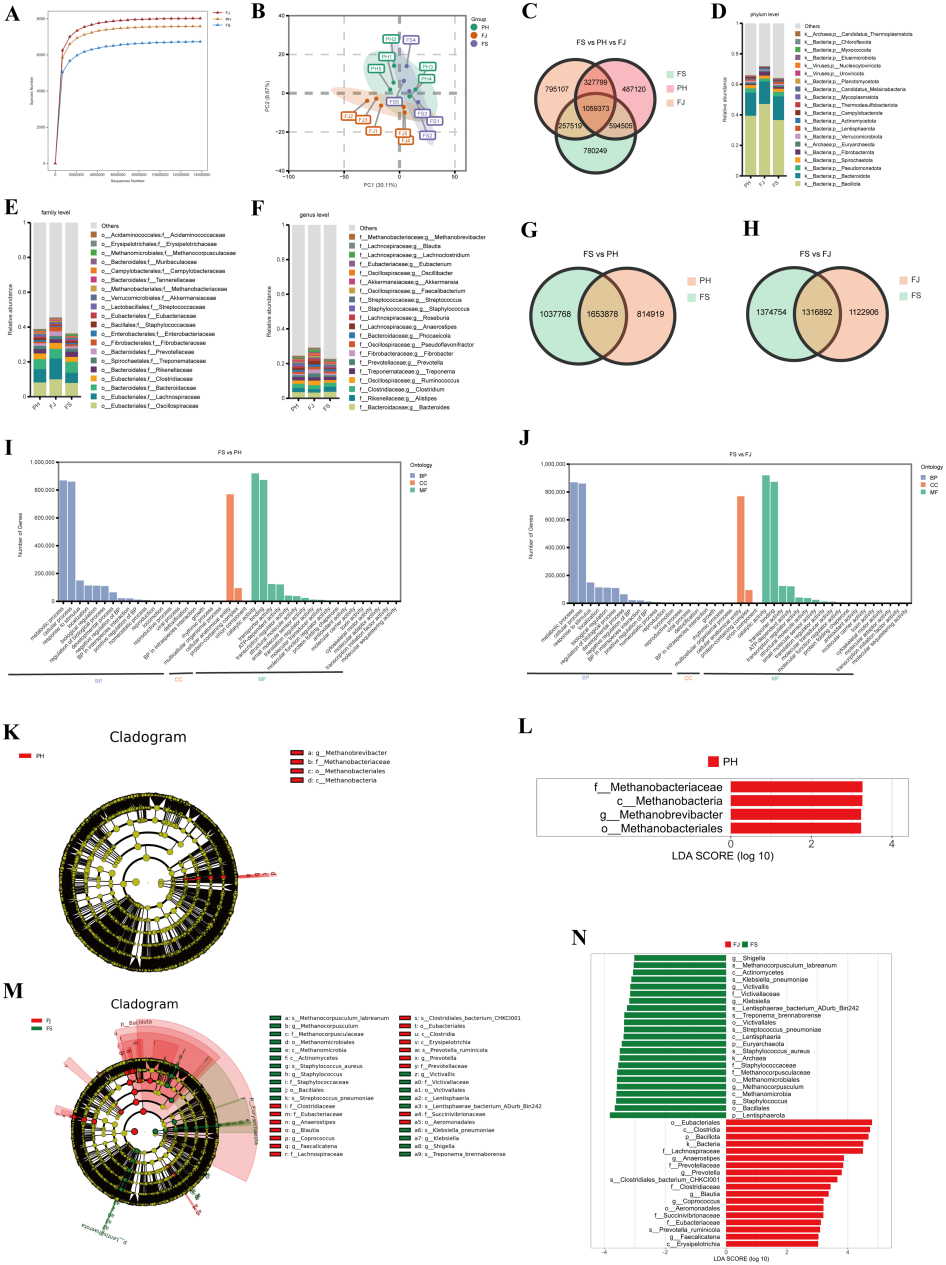
Effects of differently processed cotton stalk diets on colonic microbial community structure and functional profiling in Hu sheep. **(A)** Rarefaction curves based on metagenomic sequencing, **(B)** Principal component analysis (PCA) of microbial community composition, and **(C)** Venn diagram showing shared and unique genes among the FS, PH, and FJ groups. Relative abundance of the predominant taxa at the **(D)** phylum, **(E)** family, and **(F)** genus levels. **(G,H)** Venn diagrams showing shared and unique genes between FS vs. PH and FS vs. FJ comparisons. **(I,J)** Gene ontology (GO) functional classification of differential genes for FS vs. PH and FS vs. FJ, respectively. **(K,L)** LEfSe cladogram and corresponding LDA scores identifying taxa enriched in the PH group. **(M,N)** LEfSe cladogram and corresponding LDA scores identifying taxa enriched in the FJ group. FS = diet containing ground cotton stalks; PH = diet containing steam-exploded cotton stalks; FJ = diet containing fermented cotton stalks; *n* = 5 sheep/treatment.

The rarefaction curves of all groups approached a plateau, indicating adequate sequencing depth and sufficient coverage of microbial diversity within the colonic samples (Figure 3A). Among the three treatments, the FJ group exhibited the highest species richness, followed by PH and FS. Principal component analysis (PCA) showed clear segregation of microbial communities among the three groups (Figure 3B). Samples within each group clustered closely together, while the FJ group formed a distinct cluster separated from FS and PH along the principal components, suggesting differences in overall microbial community composition. The Venn diagram (Figure 3C) summarizes differential genes identified across the three treatments. In total, 1,059,373 differential genes were shared by all groups. Treatment-specific genes numbered 780,249 in FS, 487,120 in PH, and 795,107 in FJ, indicating a large shared core alongside distinct, processing-specific gene sets.

At the phylum level (Figure 3D), the three predominant phyla in colonic samples were *Bacillota, Bacteroidota,* and *Pseudomonadota*. The relative abundances of Bacillota in the FS, PH, and FJ groups were 0.366, 0.395, and 0.472, respectively. *Bacteroidota* accounted for 0.155, 0.152, and 0.147, and *Pseudomonadota* for 0.029, 0.028, and 0.019. At the family level (Figure 3E), the top five families were Oscillospiraceae, Lachnospiraceae, Bacteroidaceae, Clostridiaceae, and Rikenellaceae. Their relative abundances in FS, PH, and FJ were as follows: Oscillospiraceae–0.079, 0.082, and 0.100; Lachnospiraceae–0.058, 0.076, and 0.119; Bacteroidaceae–0.063, 0.058, and 0.056; Clostridiaceae–0.029, 0.033, and 0.034; and Rikenellaceae–0.030, 0.025, and 0.020. The FJ group showed higher relative abundances of Oscillospiraceae and Lachnospiraceae, families often linked with short-chain fatty acid production. At the genus level (Figure 3F), the top seven genera were *Bacteroides, Alistipes, Clostridium, Ruminococcus, Treponema, Prevotella*, and *Fibrobacter*. Their relative abundances in FS, PH, and FJ were 0.037, 0.035, 0.033 for *Bacteroides*; 0.029, 0.024, 0.020 for *Alistipes*; 0.023, 0.024, 0.023 for *Clostridium*; 0.016, 0.020, 0.026 for *Ruminococcus*; 0.018, 0.019, 0.019 for *Treponema*; 0.010, 0.011, 0.023 for *Prevotella*; and 0.010, 0.008, 0.023 for *Fibrobacter*. The number of shared genes between the FS and PH groups was 1,653,878 (Figure 3G), while the number of shared genes between the FS and FJ groups was 1,122,906 (Figure 3H).

Gene Ontology (GO) mapping for the FS vs. PH comparison showed that most annotated genes fell under Biological Process (BP) terms, led by metabolic process, cellular process, and response to stimulus, followed by localization, biological regulation, regulation of biological process (both negative and positive), developmental process, interspecies interaction, homeostatic process, and reproduction/viral process/cell death (Figure 3I). Within the Cellular Component (CC) category, genes were mainly assigned to cellular anatomical entity and protein-containing complex. For Molecular Function (MF), the most represented terms were catalytic activity and binding, with additional contributions from transporter activity, ATP-dependent activity, transcription regulator activity, structural molecule activity, small-molecule sensor activity, translocase activity, translation regulator activity, protein-folding chaperone, antioxidant activity, and molecular carrier activity. In the FS vs. FJ comparison, most annotated genes mapped to the BP terms, with the largest counts in metabolic process, cellular process, and response to stimulus, followed by localization, biological regulation, regulation of biological process (negative and positive), developmental process, interspecies interaction, homeostatic process, and reproduction (Figure 3J). Within the CC category, genes were predominantly assigned to cellular anatomical entity and protein-containing complex. For the MF, the most represented terms were catalytic activity and binding, with additional enrichment in transporter activity, ATP-dependent activity, transcription regulator activity, structural molecule activity, small-molecule sensor/transducer activity, translocase activity, translation regulator activity, protein-folding chaperone, antioxidant activity, and molecular carrier activity.

LEfSe identified PH-specific discriminative clades, while no biomarkers passed the LDA threshold for the FS group. The cladogram (Figure 3K) highlights a contiguous lineage within the archaea, comprising *c_Methanobacteria*, *o_Methanobacteriales*, *f_Methanobacteriaceae, g_Methanobrevibacter*. The bar plot (Figure 3L) shows these taxa with significant LDA scores (log10), indicating their enrichment in the PH group. LEfSe identified a set of FJ-enriched biomarkers spanning the *c_Clostridia*/*o_Eubacteriales* and the *f_Lachnospiraceae, Clostridiaceae, Eubacteriaceae*, *Prevotellaceae* and *Succinivibrionaceae* (Figures 3M,N). At finer ranks, the genera highlighted for FJ included *Anaerostipes, Blautia, Coprococcus, Faecalicatena*, and *Prevotella* (including *Prevotella ruminicola*).

### 3.5 Effects of fermented cotton stalks on microbial metabolites in the colon of sheep

**Figure 4:**
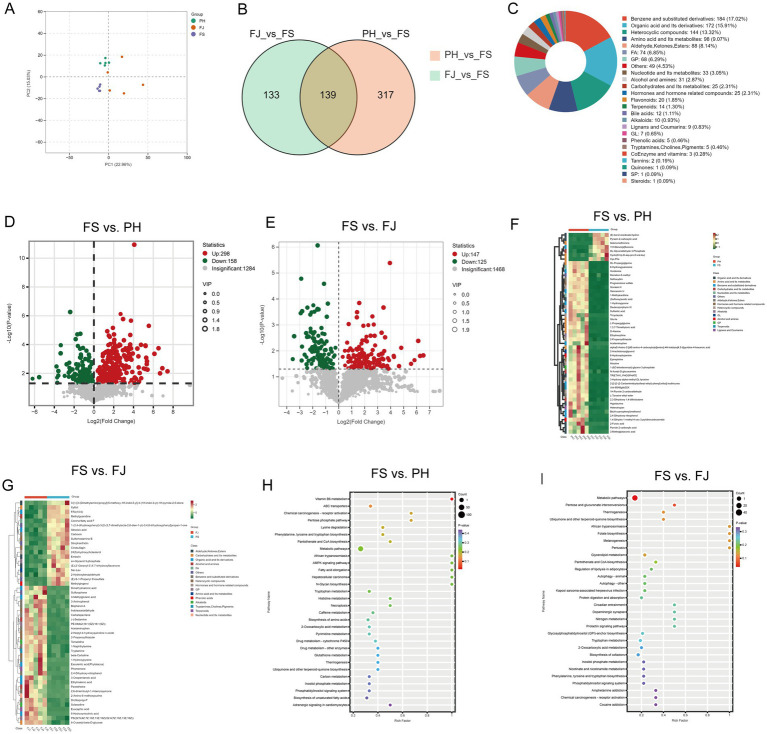
Metabolomic profiling of colonic digesta in Hu sheep fed differently processed cotton stalk diets. **(A)** PCA score plot showing separation among the FS, PH, and FJ groups. **(B)** Venn diagram summarizing the overlap of differential metabolites in the FJ_vs_FS and PH_vs_FS comparisons. **(C)** Chemical class distribution of annotated metabolites detected in colonic digesta. **(D,E)** Volcano plots of differential metabolites for FS vs. PH **(D)** and FS vs. FJ **(E)**. **(F,G)** Hierarchical clustering heatmaps of the top 50 differential metabolites for FS vs. PH **(F)** and FS vs. FJ **(G)**. **(H,I)** KEGG pathway enrichment of differential metabolites for FS vs. PH **(H)** and FS vs. FJ **(I)**. FS = diet containing ground cotton stalks; PH = diet containing steam-exploded cotton stalks; FJ = diet containing fermented cotton stalks; *n* = 5 sheep/treatment.

Metabolomic profiling of colonic digesta was conducted, and principal component analysis (PCA) revealed that the samples were mainly separated along PC1, which explained 22.96% of the variance, and PC2, which explained 15.03% of the variance (Figure 4A). Biological replicates within each treatment clustered closely together, indicating good within-group reproducibility. Compared with the FS group, samples from the FJ group were clearly shifted toward the positive side of PC1, whereas those from the PH group were located between the FS and FJ clusters. To further characterize these alterations, pairwise comparisons were performed using the FS group as the reference, and the overlap of differential metabolites was summarized in a Venn diagram (Figure 4B). In total, 272 differential metabolites were identified in the FJ_vs_FS comparison and 456 in the PH_vs_FS comparison. Among these, 139 metabolites were shared between the two comparisons, while 133 metabolites were unique to FJ_vs_FS and 317 were unique to PH_vs_FS. Based on the overall classification of metabolites across all samples (Figure 4C), benzene and substituted derivatives represented the largest category, with 184 metabolites accounting for 17.02% of the total. Organic acids and their derivatives ranked second (172 metabolites, 15.91%), followed by heterocyclic compounds (144 metabolites, 13.32%). Amino acids and their metabolites formed the fourth major group (98 metabolites, 9.07%), and aldehydes, ketones and esters constituted the fifth (88 metabolites, 8.14%).

Differential metabolite analysis between treatments is illustrated by the volcano plots (Figures 4D,E). In the comparison between PH and FS (Figure 4D), a total of 456 metabolites showed significant differences, with 298 metabolites increased and 158 decreased in the PH group relative to the FS group. Similarly, in the comparison between FJ and FS (Figure 4E), 272 metabolites were identified as differentially abundant, including 147 metabolites that were upregulated and 125 that were downregulated in the FJ group compared with the FS group.

For the FS vs. PH comparison, hierarchical clustering of the top 50 differential metabolites clearly separated the two groups and revealed opposite patterns of change.

Notably, the levels of multiple pesticide- or pollutant-like chemicals, including 11H-benzo[a]fluorene, demeton-S-methyl, sethoxydim, tricyclazole, atrazine, triethyl phosphate, 2,4-dihydroxy-nitrophenol, sulfanilic acid, 1-hydroxypyrene and Bis(4-cyanophenyl)methanol, were lower in the PH group than in the FS group. In addition, several oxidative-stress or damage markers such as 8-hydroxyguanosine, 6-hydroxydopamine and 1-methylxanthine were also decreased under PH feeding. These changes suggest that the PH diet was associated with a reduction in colonic accumulation of aromatic pollutants and oxidative stress–related metabolites. However, some potentially beneficial metabolites, including selenometionine, hypotaurine, *N*-acetyl-D-glucosamine, 2-arachidonoylglycerol, DL-glyceraldehyde-3-phosphate and small peptides such as Cys-Phe and Glu-Ile, also declined in the PH group. Conversely, metabolites that were enriched in PH but low in FS included several organic acids and bioactive plant- or microbially derived compounds. Among them, dimethylmalonic acid, 3-methylglutaric acid and 3-methylglutaconic acid are short-chain dicarboxylic acids related to intermediary energy metabolism, while sulforaphene, indoleacetaldehyde, tryptamine and esculentic acid (Phytolacca-derived) are known representatives of isothiocyanates, indole derivatives and phytochemicals. At the same time, the PH group also showed higher levels of several xenobiotic-like or aromatic compounds, such as bisphenol A, 1-naphthylamine, 3-aminophenol, 2-heptyl-4-hydroxyquinoline N-oxide and acetaminophen (Figure 4F). In the FS vs. FJ comparison, the FJ group was characterized by a metabolite cluster that was consistently elevated relative to FS and was mainly composed of compounds with potential barrier-protective or anti-inflammatory properties. Specifically, FJ-fed sheep showed higher levels of the sugar alcohol xylitol and the medium-chain free fatty acid FFA(10:0), both of which are related to microbial carbohydrate and lipid metabolism and may contribute to local energy supply for epithelial cells. Several plant- or microbially derived signaling molecules were also enriched in the FJ group, including abscisic acid, methylgingerol, Embelin, sulforaphene, and the flavanone derivative (E)-2′-geranyl-3′,4′,7-trihydroxyflavanone. In addition, FJ samples contained higher concentrations of multiple organic acids and dicarboxylic acids, such as dimethylmalonic acid, 3-methylglutaric acid, 3-methylglutaconic acid, ethylmalonic acid, and 3-oxopentanoic acid, together with sn-glycerol-3-phosphate,6-O-acetyl-*β*-D-glucose, indoleacetaldehyde and tryptamine (Figure 4G).

In the FS vs. PH comparison, KEGG enrichment indicated that the PH diet activated several pathways beneficial for epithelial homeostasis, including vitamin B6 metabolism, pentose phosphate pathway, 2-oxocarboxylic acid metabolism, fatty acid elongation, biosynthesis of amino acids, glutathione metabolism and AMPK signaling. These changes are consistent with the higher levels of short-chain organic acids (dimethylmalonic acid, 3-methylglutaric acid, 3-methylglutaconic acid) and tryptophan-derived indole metabolites (indoleacetaldehyde, tryptamine) in the PH group, suggesting improved energy supply and antioxidant capacity at the mucosal surface. However, PH also enriched pathways related to xenobiotic and injury responses, such as drug metabolism–cytochrome P450, chemical carcinogenesis–receptor activation and necroptosis, which parallel the accumulation of bisphenol A, 1-naphthylamine, 3-aminophenol, 1-hydroxypyrene and acetaminophen. Thus, PH fermentation confers partial metabolic benefits for the colonic barrier but maintains a noticeable xenobiotic burden (Figure 4H). In the FS vs. FJ comparison, KEGG enrichment showed that FJ feeding mainly enhanced pathways related to tryptophan metabolism, phenylalanine/tyrosine/tryptophan biosynthesis, 2-oxocarboxylic acid metabolism, nicotinate and nicotinamide metabolism, inositol phosphate/phosphatidylinositol signaling, and autophagy (Figure 4I).

### 3.6 Effects of processed cotton stalk diets on colonic inflammatory cytokines

**Figure 5:**
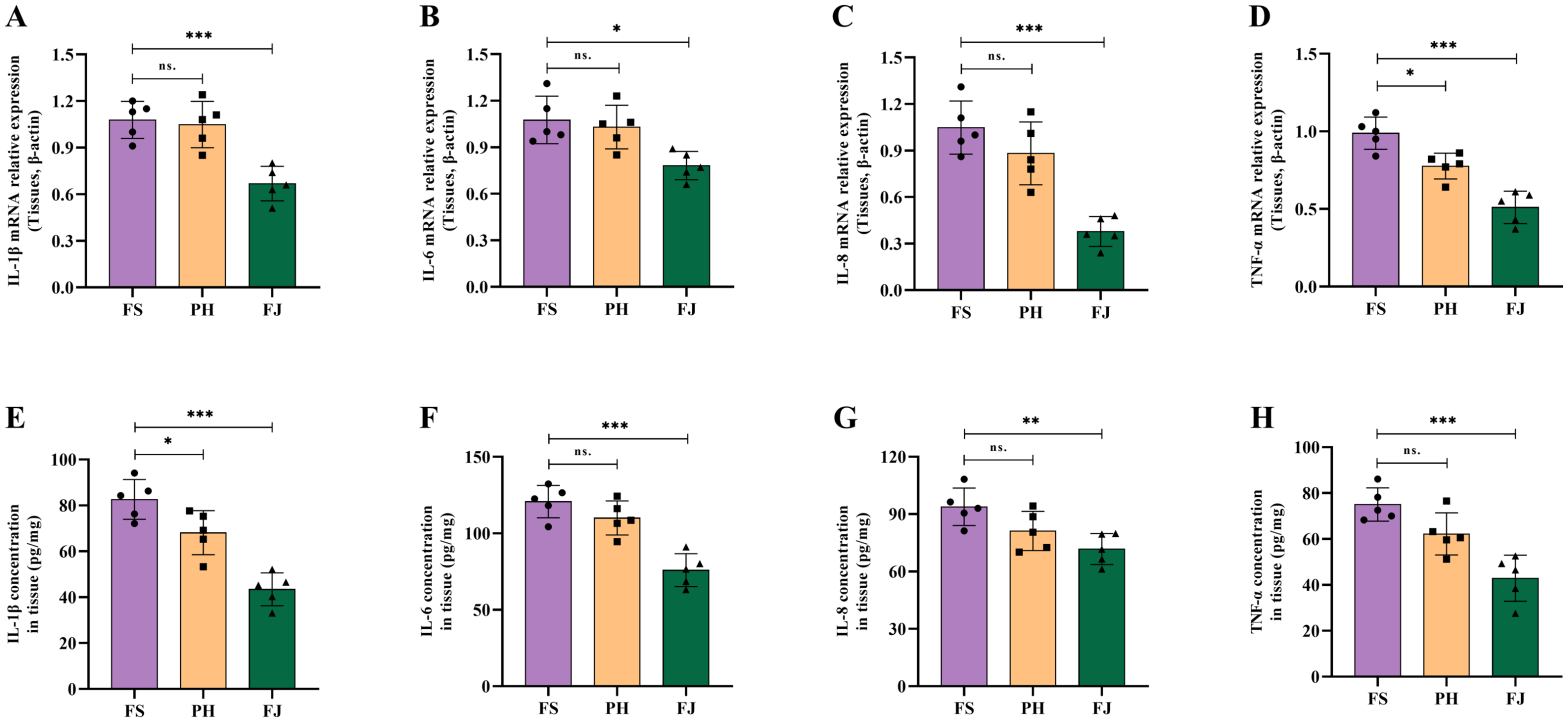
Fermented cotton stalks attenuate colonic inflammatory responses in Hu sheep. **(A–D)** Relative mRNA expression of pro-inflammatory cytokines in colonic tissues, including IL-1*β* **(A)**, IL-6 **(B)**, IL-8 **(C)**, and TNF-α **(D)**, normalized to β-actin. **(E–H)** Tissue concentrations of IL-1β **(E)**, IL-6 **(F)**, IL-8 **(G)**, and TNF-α **(H)** in the colon. FS = diet containing ground cotton stalks; PH = diet containing steam-exploded cotton stalks; FJ = diet containing fermented cotton stalks; *n* = 5 sheep/treatment. Data are presented as means ± SEM. Statistical differences among treatments are indicated by *p < 0.05, **p < 0.01, ***p < 0.001; ns, not significant.

At the mRNA level, dietary treatment markedly affected the expression of pro-inflammatory cytokines in the colonic mucosa (Figures 5A–D). Compared with the FS group, the PH group showed no significant changes in IL-1β, IL-6 or IL-8 mRNA expression, but TNF-*α* mRNA was significantly reduced (*p* < 0.05). In contrast, the FJ group exhibited significantly lower mRNA expression of IL-1β, IL-6, IL-8 and TNF-α than the FS group (*p* < 0.05), indicating that feeding fermented cotton stalks suppresses colonic mucosal pro-inflammatory cytokine expression at the transcriptional level.

Consistent with the qPCR results, ELISA measurements showed a similar pattern for cytokine concentrations in colonic mucosal tissue (Figures 5E–H). Relative to the FS group, the PH group displayed a significant decrease in IL-1β content (*p* < 0.05), whereas IL-6, IL-8 and TNF-α concentrations did not differ significantly. By contrast, the FJ group had significantly lower tissue concentrations of IL-1β, IL-6, IL-8 and TNF-α than the FS group (*p* < 0.01).

### 3.7 Effects of processed cotton stalk diets on colonic NF-κB/MLCK pathway and tight junction proteins

**Figure 6:**
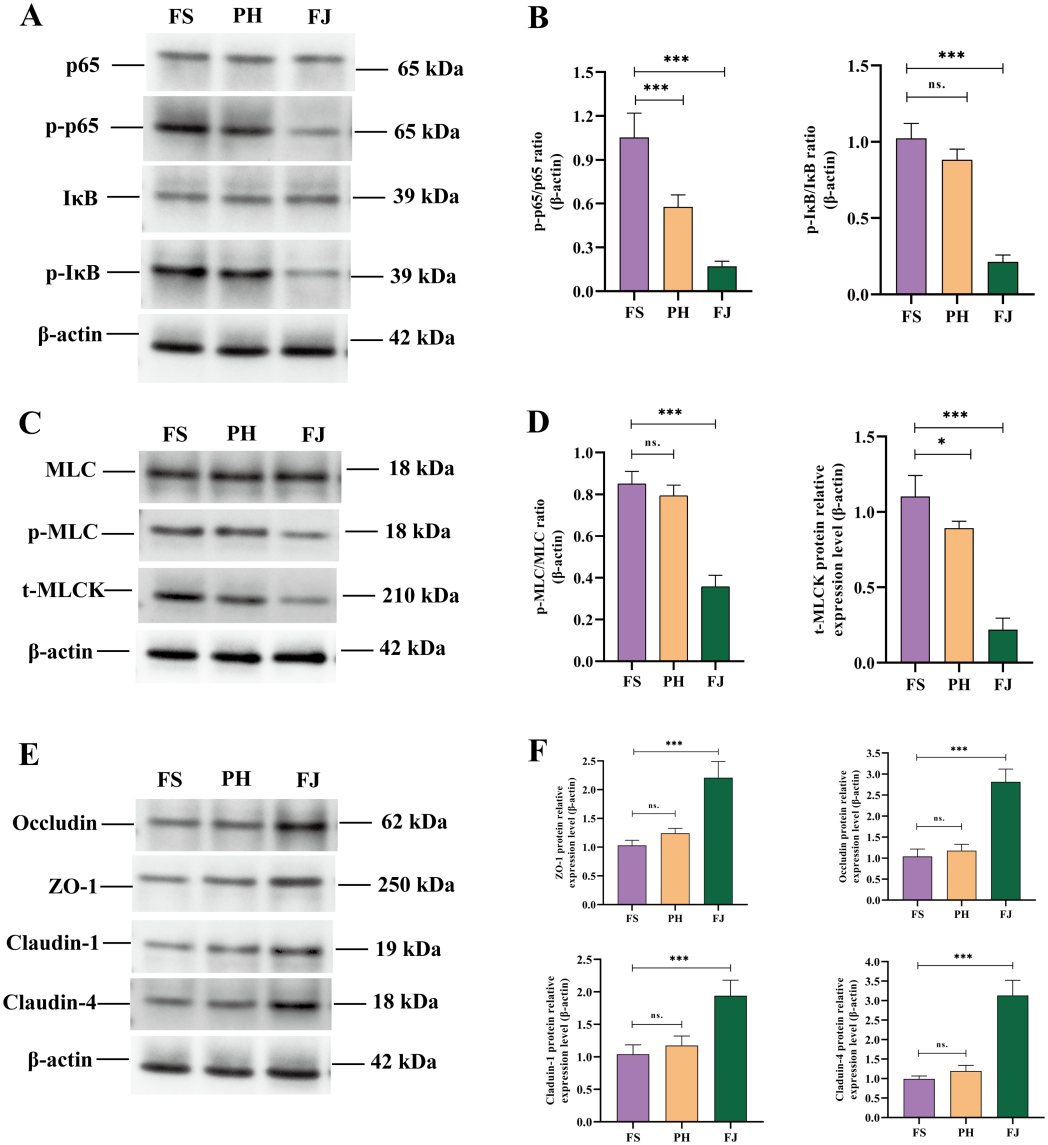
Fermented cotton stalks inhibit NF-κB/MLCK signaling and enhance tight-junction protein abundance in the colonic mucosa of Hu sheep. **(A)** Representative western blot bands of p65, phosphorylated p65 (p-p65), IκB, p-IκB, and β-actin in colonic tissues from the FS, PH, and FJ groups. **(B)** Densitometric quantification of p-p65/p65 and p-IκB/IκB ratios. **(C)** Representative western blot bands of MLC, p-MLC, t-MLCK, and β-actin. **(D)** Densitometric quantification of p-MLC/MLC ratio and t-MLCK protein level. **(E)** Representative western blot bands of tight-junction proteins (occludin, ZO-1, claudin-1, and claudin-4) and β-actin. **(F)** Densitometric quantification of ZO-1, occludin, claudin-1, and claudin-4 protein levels normalized to β-actin. FS = diet containing ground cotton stalks; PH = diet containing steam-exploded cotton stalks; FJ = diet containing fermented cotton stalks; *n* = 5 sheep/treatment. Data are presented as means ± SEM. Statistical differences among treatments are indicated by *p < 0.05, **p < 0.01, ***p < 0.001; ns, not significant.

Feeding fermented cotton stalks strongly modulated the colonic NF-κB/MLCK signaling pathway and tight junction proteins (Figure 6). At the level of NF-κB activation (Figures 6A,B), the FS group showed the highest p-p65/p65 ratio. Compared with the FS group, the PH group exhibited a significantly lower p-p65/p65 ratio (*p* < 0.001), and the FJ group showed an even greater reduction (*p* < 0.001). For IκB phosphorylation, the p-IκB/IκB ratio in the PH group did not differ from that in the FS group (*p* > 0.05), whereas the FJ group displayed a marked decrease compared with the FS group (*p* < 0.001).

Consistent with reduced NF-κB activation, FJ feeding also decreased downstream MLCK signaling (Figures 6C,D). Compared with the FS group, the PH group showed no significant change in the p-MLC/MLC ratio (*p* > 0.05), whereas the FJ group had a significantly lower p-MLC/MLC ratio (*p* < 0.001). In addition, total MLCK protein abundance was slightly decreased in the PH group compared with the FS group (*p* < 0.05) and was markedly decreased in the FJ group (*p* < 0.001).

Tight junction–associated proteins showed the opposite pattern (Figures 6E,F). Compared with the FS group, the PH diet did not significantly affect the protein levels of ZO-1, occludin, claudin-1 or claudin-4 (*p* > 0.05). In contrast, the FJ diet markedly increased all four tight junction proteins, with ZO-1, occludin, claudin-1 and claudin-4 protein expression being significantly higher in the FJ group than in the FS group (*p* < 0.001).

## 4 Discussion

This study demonstrates that processing cotton stalk as a roughage source can improve animal performance and mitigate adverse hindgut responses that are often associated with low-quality, high-fiber crop residues. Compared with the FS diet, both PH and FJ altered colonic fermentation, microbial ecology, and mucosal signaling; however, the fermented cotton stalk produced the most internally consistent phenotype—improved feed efficiency, higher propionate and butyrate, lower colonic NH3-N, enrichment of taxa correlated with butyrate production, and reinforcement of tight-junction integrity with reduced inflammatory activation. Collectively, these data support a mechanistic link between feed processing, hindgut metabolite supply, and barrier regulation.

Cotton stalk is an attractive feed resource in regions with substantial cotton production, but its use is constrained by high lignin content and the presence of free gossypol and other field residues (17). These factors can limit intake and nutrient utilization and may increase systemic exposure to free gossypol (10). In sheep, increasing dietary cotton stalk can raise plasma and tissue free gossypol concentrations and impair growth when dietary free gossypol levels exceed common safety thresholds (18, 49). The improvement in ADG and the reduction in feed-to-gain ratio observed with processed cotton stalk, particularly in the FJ group, therefore suggests that processing reduced at least one of the major constraints on cotton stalk utilization. Steam explosion is widely used to disrupt the lignocellulosic matrix and improve microbial accessibility of cellulose by partially solubilizing hemicellulose and increasing surface area (19–21). In animal nutrition contexts, steam explosion of crop residues has been reported to improve *in vitro* ruminal fermentation and energy utilization (22, 23). Fermentation can further improve feed value by generating organic acids, providing exogenous fibrolytic enzymes, partially hydrolyzing complex polymers, and reducing anti-nutritional factors (24). For cotton byproducts, microbial fermentation is an effective strategy for decreasing free gossypol and improving nutrient profiles, with Bacillus-based solid-state fermentation being particularly effective in gossypol removal (4, 25). The superior feed conversion in the FJ group is consistent with these combined effects: enhanced substrate availability coupled with lower exposure to compounds that can suppress performance.

Although the rumen is the primary site of microbial fermentation, hindgut fermentation can contribute approximately 5–10% of dietary energy in ruminants and becomes increasingly important when fermentable substrates escape foregut digestion (26, 27). In the present study, FJ decreased colonic pH while increasing total VFA, with a shift toward higher propionate and butyrate and a concomitant reduction in NH_3_-N. These changes are compatible with a greater proportion of luminal fermentation being driven by carbohydrate rather than protein. Lower NH_3_-N in particular suggests reduced proteolysis/deamination or improved microbial capture of nitrogen in the hindgut, both of which are generally interpreted as favorable for epithelial health (28). Among VFAs, butyrate is of special relevance for the large intestine because it is a preferred oxidative substrate for colonocytes and directly supports barrier maintenance and repair (29). In epithelial models, butyrate enhances barrier function by increasing transepithelial resistance, tightening paracellular flux, and upregulating tight-junction proteins such as claudin-1 (30). Propionate and acetate can also influence epithelial and immune physiology through GPCR signaling and as metabolic substrates; more broadly, SCFAs are recognized as major microbiota-derived signaling molecules that connect fiber fermentation to host physiology (31). Therefore, the higher propionate and butyrate observed in FJ provide a plausible upstream driver for the downstream improvements in mucosal morphology and tight-junction protein expression. Consistent with this SCFA-centered interpretation, the untargeted metabolomics further pointed to diet-sensitive microbial signaling candidates (including tryptophan-derived indole/amine metabolites and lipid mediators), which provides additional pathway-relevant context for the downstream barrier phenotype.

The microbial community in the colon adapted to cotton stalk processing. While Bacillota and Bacteroidota dominated across diets, FJ was associated with shifts in genera that are frequently linked to butyrate production and cross-feeding networks. Taxa within *Lachnospiraceae* and related groups (e.g., *Anaerostipes*, *Blautia*, *Coprococcus*) are commonly reported as butyrate-associated organisms and are often enriched in conditions with higher fermentable fiber availability. Butyrate formation in these systems typically relies on cross-feeding, including utilization of lactate and acetate produced by other organisms. Lactate-utilizing butyrate producers have been isolated and characterized in classical fermentation studies, highlighting the metabolic basis for this guild structure (32). In the present study, the positive correlations among FJ-associated taxa and butyrate concentrations are consistent with this ecological framework. An additional observation was that strong discriminatory clades were detected for FS and PH, while FJ did not present a single dominant biomarker. In complex microbial ecosystems, the absence of a sharp biomarker signature can be interpreted as a more even community with higher functional redundancy, which may improve resilience to dietary perturbation. This is relevant for ruminant hindgut health, because abrupt increases in fermentable substrate supply can provoke hindgut acidosis, compromise epithelial integrity, and alter microbial communities (33, 34). Thus, the combination of higher butyrate and a more evenly structured community in FJ provides a coherent ecological explanation for the improved barrier phenotype.

Free gossypol remains a central concern for cotton-derived feeds. Although rumen microbes can detoxify a substantial portion, sheep feeding trials demonstrate that systemic exposure and tissue retention still rise with dietary cotton stalk intake, and high exposure can reduce weight gain and alter blood indices (3, 18). In the current work, the reduced free gossypol burden associated with fermented cotton stalk is therefore biologically meaningful and may contribute both to improved performance and to reduced mucosal stress. Fermentation has a documented capacity to reduce free gossypol in cotton byproducts. *Bacillus coagulans*–based solid-state fermentation can decrease free gossypol content dramatically while improving protein metrics and generating high viable/spore counts (35). Similarly, recent work on cotton residue fermentation indicates that solid-state fermentation can improve nutrient composition while reducing free gossypol (36). These studies provide mechanistic context for why fermented cotton stalk could produce a more favorable hindgut environment: lower exposure to a redox-active toxin coupled with improved fermentability that supports beneficial metabolite production. The metabolomic shifts observed here also suggest that xenobiotic-like compounds differed among diets. Cotton stalk can carry pesticide or plant growth regulator residues depending on agronomic practice, and this has been flagged as an additional risk in animal feeding contexts (37). The present dataset does not allow attribution of these differences to direct microbial degradation versus altered release from plant matrices or altered intestinal retention. However, in linking metabolomics to the core pathway, we place greater weight on metabolites with established microbial origin or microbiota–host signaling relevance (e.g., SCFAs quantified here and other signaling candidates highlighted by the metabolite profile) than on broad xenobiotic annotations. Nonetheless, the parallel reduction in inflammatory signaling and improved tight-junction expression in FJ is consistent with a lower luminal burden of compounds that can trigger epithelial stress.

The ruminant hindgut epithelium is a single-layer barrier and is therefore susceptible to damage from organic acid accumulation and luminal toxins. High concentrate feeding can induce mucosal injury and inflammatory activation in goat hindgut, including increased NF-κB protein abundance (15, 38). More broadly, models of hindgut acidosis demonstrate that lowered hindgut pH can impair epithelial integrity and increase permeability (39, 40). Against this background, the present findings that FJ improved colonic morphology, increased tight-junction protein abundance (ZO-1, occludin, claudin-1/4), and reduced inflammatory cytokine expression indicate that processed cotton stalk, and particularly fermentation, alleviated epithelial stress rather than exacerbating it. The NF-κB/MLCK axis offers a mechanistic bridge between luminal cues and paracellular permeability. In intestinal epithelial systems, inflammatory stimuli such as TNF-*α* can activate canonical NF-κB signaling to induce MLCK gene expression, leading to cytoskeletal contraction and increased tight-junction permeability (41, 42). Consequently, the reduced NF-κB activation and lower MLCK expression observed in FJ are consistent with a barrier-protective state. This signaling phenotype aligns with the metabolite changes: butyrate can inhibit NF-κB–dependent inflammatory responses in colonic tissues (43) and can directly enhance transcription and organization of tight-junction components (30). Therefore, increased butyrate availability provides a plausible upstream driver of reduced inflammatory activation and improved junctional integrity.

Mechanistically, butyrate is recognized not only as an energy substrate but also as a signaling molecule. It functions as a histone deacetylase (HDAC) inhibitor, thereby suppressing NF-κB activation through epigenetic regulation of pro-inflammatory gene transcription (44). In addition, butyrate can activate G-protein–coupled receptors, including GPR41, GPR43, and GPR109A, which are implicated in anti-inflammatory signaling and maintenance of epithelial barrier integrity (45, 46). Activation of these pathways has been shown to attenuate NF-κB signaling and modulate downstream targets involved in tight-junction regulation. Thus, the elevated butyrate concentration in the FJ group may contribute to suppression of the NF-κB/MLCK axis via both HDAC-dependent epigenetic mechanisms and receptor-mediated signaling pathways.

In addition to SCFAs, microbial tryptophan catabolites are increasingly recognized as regulators of mucosal immunity and epithelial homeostasis. Microbiota-derived tryptophan catabolites can engage the aryl hydrocarbon receptor and shape mucosal reactivity via IL-22–dependent pathways (47). The enrichment of indole-related metabolites in the FJ group is consistent with this signaling axis and provides an additional mechanistic layer that could complement SCFA-mediated effects. While the present work did not directly measure AhR activation or IL-22 signaling, the metabolite profile supports the hypothesis that fermentation altered aromatic amino acid metabolism in a direction associated with improved mucosal resilience.

However, this study has several limitations, including a modest sample size and terminal sampling, which precludes characterization of temporal dynamics. Even so, the evidence was internally consistent across performance, fermentation, microbiota, and mucosal signaling readouts. Fermentation-based processing of cotton stalk improved growth performance and promoted a hindgut metabolic profile characterized by higher propionate and butyrate, accompanied by microbiota remodeling consistent with butyrogenic cross-feeding and enhanced colonic barrier integrity with reduced NF-κB/MLCK-associated inflammatory activation. These results support the practical use of fermented cotton stalk to valorize cotton agricultural residues as ruminant feed while mitigating gossypol-related and other luminal stressors.

## 5 Conclusion

**Figure 7:**
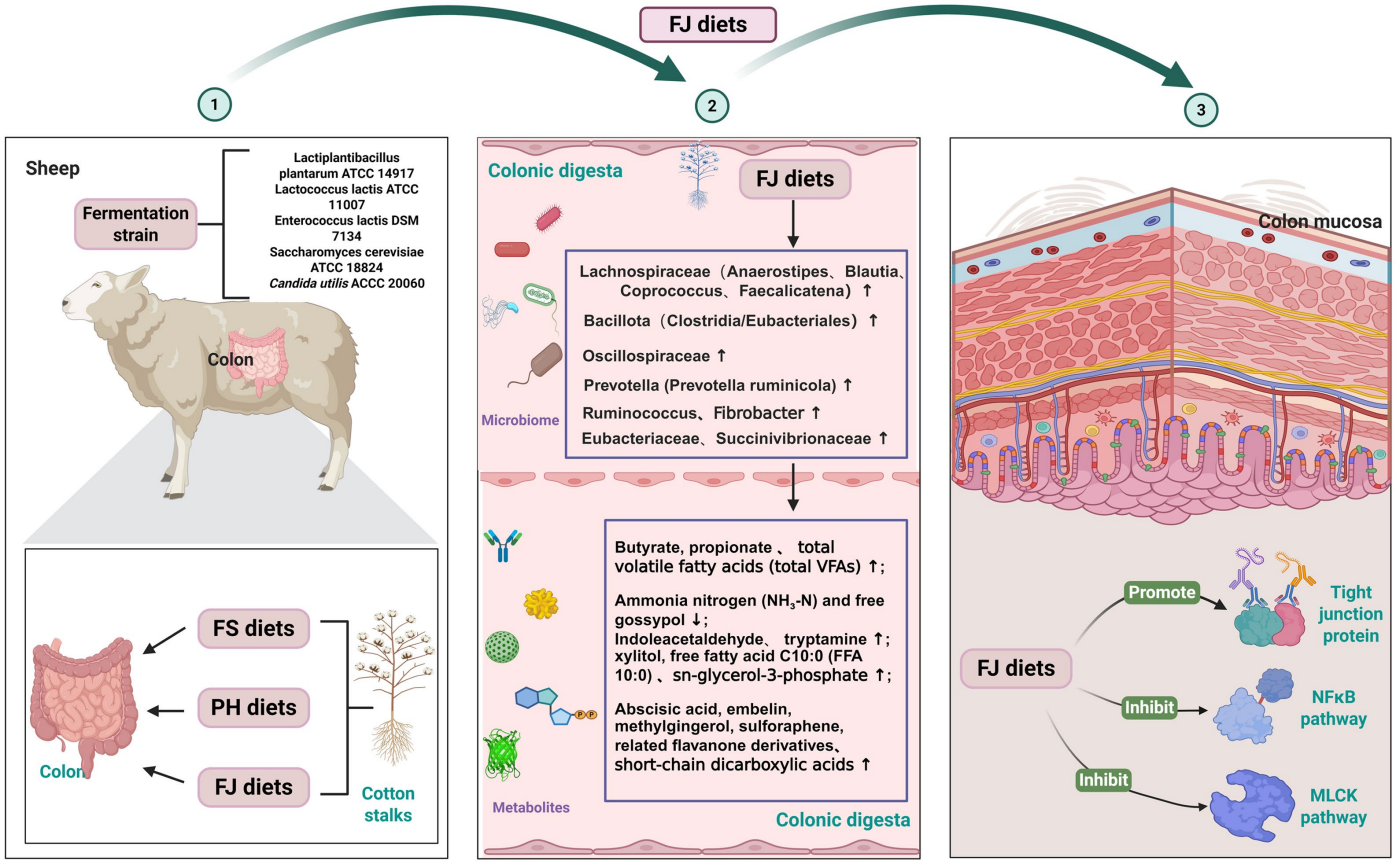
Proposed mechanism by which fermented cotton stalk diets improve hindgut microecology and reinforce colonic barrier function in Hu sheep. Schematic overview of the working model linking fermented cotton stalk (FJ) feeding to changes in colonic microbiota, metabolite profiles, and mucosal barrier regulation. (1) Experimental design illustrating the three dietary treatments based on differently processed cotton stalks (FS, PH, and FJ) and the fermentation consortium used for cotton stalk fermentation. (2) FJ diets reshape the colonic microbial community, characterized by enrichment of fiber- and butyrate-associated taxa (e.g., *Lachnospiraceae*-related genera such as *Anaerostipes*, *Blautia*, *Coprococcus* and *Faecalicatena*), together with increased propionate, butyrate, and total volatile fatty acids, and reduced ammonia nitrogen and free gossypol in colonic digesta. Key differential metabolites identified by metabolomics are highlighted. (3) These microbiota–metabolite alterations are proposed to promote tight-junction protein expression while inhibiting NF-κB activation and downstream MLCK signaling, thereby maintaining colonic mucosal integrity. FS = diet containing ground cotton stalks; PH = diet containing steam-exploded cotton stalks; FJ = diet containing fermented cotton stalks.

In general, the results of the present study indicate that direct feeding of cotton stalks can compromise colonic epithelial integrity and promote mucosal inflammatory activation in Hu sheep, whereas fermentation-based processing effectively reverses these adverse responses (Figure 7). The protective effect of fermented cotton stalks is characterized by a shift in hindgut fermentation toward higher propionate and butyrate with reduced NH₃–N and free gossypol exposure, accompanied by microbiota remodeling consistent with butyrogenic cross-feeding and reinforcement of tight-junction integrity. Importantly, these benefits are mechanistically aligned with attenuation of NF-κB activation and suppression of the downstream MLCK–p-MLC pathway, thereby limiting cytokine-associated barrier disruption. Collectively, these findings support fermented cotton stalk as a practical and mechanistically grounded strategy to valorize cotton agricultural residues as ruminant roughage while mitigating gossypol-related and other luminal stressors that threaten hindgut health.

## Data Availability

The metabolomics data presented in the study are deposited in the EBI MetaboLights repository, accession number MTBLS14078; the metagenomics data presented in the study are deposited in the NCBI SRA repository, accession number PRJNA1439621.
